# OA11 A Case of Hemophagocytic Lymphohistiocytosis (HLH) Triggered by Dengue Fever in a 26-Year-Old Woman Returning from Bali: Diagnostic Challenges and Multidisciplinary Management

**DOI:** 10.1093/rap/rkae117.011

**Published:** 2024-11-05

**Authors:** Tanjina Nasrin, Zehra Gul, Ann Scott Russell

**Affiliations:** Queen Alexandra Hospital, Portsmouth, United Kingdom; Queen Alexandra Hospital, Portsmouth, United Kingdom; Queen Alexandra Hospital, Portsmouth, United Kingdom

## Abstract

**Introduction:**

This case report presents a 26-year-old woman who recently returned from Bali and developed severe systemic symptoms, including fever, rash, joint pain, and gastrointestinal disturbances. Notably, her presentation includes clinical and laboratory features suggestive of Hemophagocytic Lymphohistiocytosis (HLH), a rare but life-threatening syndrome often triggered by infections. Given her travel history and exposure to mosquito bites, the differential diagnosis included Dengue fever and other tropical infections. This case highlights the complexities of diagnosing and managing HLH in the context of travel-related infectious diseases, underscoring the importance of a multidisciplinary approach and thorough diagnostic evaluation.

**Case description:**

The patient, a 26-year-old woman with a recent travel history to Bali (April 5th to April 23rd, 2024), presented with a one-week history of fever, headache, and a rash on her lower limbs initially presumed to be sunburn. She also experienced fleeting arthralgia, nausea, diarrhoea, and a general feeling of being unwell. During her trip, she sustained superficial abrasions to her chest and reported numerous mosquito bites. Her past medical history includes migraines and endometriosis, and her regular medications are Naproxen, OCP, Fexofenadine, and a calcium supplement. She denies new sexual partners or illicit drug use and has no relevant family history.

On presentation, notable findings included red, blistering skin on her face, legs, and upper chest, sparing areas covered by her bathing suit and surfboard strap. Laboratory investigations on April 29th, 2024, revealed significant abnormalities: low white blood cell count, neutrophils, and thrombocytopenia (52), elevated liver function tests (ALT and AST > 540), ferritin 16079, raised LDH, triglycerides 1.72, and low fibrinogen (2.5). She had a high HLH score of 167, indicating a 40-54% probability of HLH. Blood and urine cultures, along with serologies for Hepatitis (A/B/C/E), HIV, EBV, and CMV, were negative. Imaging (CT CAP and X-ray) was unremarkable.

Initial management included pulse IV Methylprednisolone (1 g daily for 3 doses), followed by oral prednisolone (50 mg daily), and Anakinra (100 mg BID with standard antimicrobial prophylaxis, then tapered). Given the high suspicion of HLH, consultations with the HLH MDT team led to additional testing for Dengue and Typhoid, with a plan to adjust immunosuppressive and antimicrobial therapies based on these results. A bone marrow biopsy performed on April 30th, 2024, confirmed the diagnosis

**Discussion:**

Hemophagocytic Lymphohistiocytosis is a rare, potentially fatal hyper-inflammatory and hemophagocytic syndrome causing severe hypercytokinemia with excessive activation of lymphocytes and macrophages. It affects all ages and has no predilection for race or sex. HLH can be classified into genetic and acquired (secondary) forms, the latter associated with conditions such as infections, autoimmune diseases, and malignancies. The diagnosis of HLH is challenging due to the overlap of clinical features with other conditions.

Our patient, a 26-year-old woman, presented with fever, headache, rash, fleeting arthralgia, nausea, diarrhoea, and general malaise, initially presumed as sunburn. She had recently returned from Bali and reported numerous mosquito bites. Laboratory findings included low white blood cell count, neutropenia, thrombocytopenia, elevated liver enzymes, hyperferritinemia, and raised triglycerides. Despite negative tests for hepatitis, human immunodeficiency virus, Epstein-Barr virus, cytomegalovirus, and blood cultures, Escherichia coli was found in her urine. Given her clinical presentation and high HLH score 167, she was diagnosed with HLH, possibly triggered by dengue infection, confirmed by positive Dengue serology.

Dengue fever, caused by the Dengue virus and transmitted by Aedes mosquitoes, presents a spectrum ranging from undifferentiated fever to dengue haemorrhagic fever and expanded dengue syndrome. Typical dengue fever symptoms include severe headache, myalgia, arthralgia, and rashes, often accompanied by leukopenia and thrombocytopenia. Dengue haemorrhagic fever includes high fever, signs similar to dengue fever, and plasma leakage leading to hypovolemic shock (dengue shock syndrome). Expanded dengue syndromes encompass atypical and unusual manifestations.

Treatment for our patient included pulse intravenous Methylprednisolone followed by oral prednisolone and Anakinra, with antimicrobial prophylaxis. Her condition improved with treatment, and daily monitoring showed no relapse, allowing for a gradual tapering of Anakinra and prednisolone. This case highlights the importance of considering HLH in patients with severe dengue and persistent fever, particularly in those with travel histories to endemic areas

**Key learning points:**

• Early Recognition and Diagnosis: Hemophagocytic Lymphohistiocytosis requires prompt identification, particularly in patients with systemic inflammatory symptoms and recent travel to endemic regions. Clinicians should maintain a high index of suspicion for HLH and consider travel history as a crucial component of the diagnostic workup.

• Multidisciplinary Collaboration: Managing HLH effectively necessitates a multidisciplinary approach involving specialists from rheumatology, haematology, and infectious diseases. Collaboration ensures comprehensive evaluation, accurate diagnosis, and appropriate therapeutic interventions tailored to each patient’s needs.

• Role of Travel History : A detailed travel history is pivotal in diagnosing HLH, aiding in the identification of potential infectious triggers such as Dengue fever. Clinicians should inquire about recent travel and potential exposures to endemic diseases to guide diagnostic investigations effectively.

• Diagnostic Challenges and Investigations: HLH diagnosis relies on a combination of clinical criteria and laboratory findings, including elevated ferritin levels, pancytopenia’s, and liver dysfunction. Bone marrow biopsy and specific serologies for infectious triggers are essential for confirming the diagnosis and initiating appropriate treatment.

• Therapeutic Management: Immediate initiation of immunosuppressive therapy, such as corticosteroids and cytokine inhibitors like Anakinra, is crucial for controlling the hyperinflammatory state in HLH. Treatment plans should be individualised, with close monitoring and adjustments based on clinical response and laboratory evaluations.

• Monitoring and Follow-Up: Continuous monitoring of inflammatory markers and clinical symptoms is essential for assessing treatment efficacy and preventing relapses in HLH. Regular follow-up appointments allow for adjustments in therapy, ensuring optimal disease management and patient outcomes.

These key learning points underscore the importance of early recognition, multidisciplinary collaboration, and comprehensive management strategies in improving outcomes for patients with HLH. By adopting a structured approach to diagnosis, treatment, and follow-up, clinicians can effectively address the complexities of this rare but potentially life-threatening condition, particularly in the context of travel-related infections

